# Smart Devices for Older Adults Managing Chronic Disease: A Scoping Review

**DOI:** 10.2196/mhealth.7141

**Published:** 2017-05-23

**Authors:** Ben YB Kim, Joon Lee

**Affiliations:** ^1^ Health Data Science Lab School of Public Health and Health Systems University of Waterloo Waterloo, ON Canada

**Keywords:** mobile health, mHealth, smartphone, mobile phone, tablet, older adults, seniors, chronic disease, chronic disease management, scoping review

## Abstract

**Background:**

The emergence of smartphones and tablets featuring vastly advancing functionalities (eg, sensors, computing power, interactivity) has transformed the way mHealth interventions support chronic disease management for older adults. Baby boomers have begun to widely adopt smart devices and have expressed their desire to incorporate technologies into their chronic care. Although smart devices are actively used in research, little is known about the extent, characteristics, and range of smart device-based interventions.

**Objective:**

We conducted a scoping review to (1) understand the nature, extent, and range of smart device-based research activities, (2) identify the limitations of the current research and knowledge gap, and (3) recommend future research directions.

**Methods:**

We used the Arksey and O’Malley framework to conduct a scoping review. We identified relevant studies from MEDLINE, Embase, CINAHL, and Web of Science databases using search terms related to mobile health, chronic disease, and older adults. Selected studies used smart devices, sampled older adults, and were published in 2010 or after. The exclusion criteria were sole reliance on text messaging (short message service, SMS) or interactive voice response, validation of an electronic version of a questionnaire, postoperative monitoring, and evaluation of usability. We reviewed references. We charted quantitative data and analyzed qualitative studies using thematic synthesis. To collate and summarize the data, we used the chronic care model.

**Results:**

A total of 51 articles met the eligibility criteria. Research activity increased steeply in 2014 (17/51, 33%) and preexperimental design predominated (16/50, 32%). Diabetes (16/46, 35%) and heart failure management (9/46, 20%) were most frequently studied. We identified diversity and heterogeneity in the collection of biometrics and patient-reported outcome measures within and between chronic diseases. Across studies, we found 8 self-management supporting strategies and 4 distinct communication channels for supporting the decision-making process. In particular, self-monitoring (38/40, 95%), automated feedback (15/40, 38%), and patient education (13/40, 38%) were commonly used as self-management support strategies. Of the 23 studies that implemented decision support strategies, clinical decision making was delegated to patients in 10 studies (43%). The impact on patient outcomes was consistent with studies that used cellular phones. Patients with heart failure and asthma reported improved quality of life. Qualitative analysis yielded 2 themes of facilitating technology adoption for older adults and 3 themes of barriers.

**Conclusions:**

Limitations of current research included a lack of gerontological focus, dominance of preexperimental design, narrow research scope, inadequate support for participants, and insufficient evidence for clinical outcome. Recommendations for future research include generating evidence for smart device-based programs, using patient-generated data for advanced data mining techniques, validating patient decision support systems, and expanding mHealth practice through innovative technologies.

## Introduction

The advent of the cellular phone and its wide adoption by the general public intrigued many medical and health researchers. mHealth—the delivery of health care services and information via mobile technologies—has provided unique benefits to fulfill these needs because they are portable, affordable, widely available, and widely adopted [1-3]. mHealth research with cellular phones for chronic disease management has been extensive, and many systematic reviews have concluded that this technology has had a positive but limited impact on clinical outcomes, including improved hemoglobin A_1c_ (HbA_1c_) control for people with diabetes [2,4,5] and tighter blood pressure control among hypertensive patients [6-8].

In the last few years, the boundaries of mHealth have been further expanded, driven by the rapid advancements in technological capabilities through the integration of an array of sensors (eg, accelerometer, pulse oximeter), multitouch screens, and higher computing power [9]. Most importantly, smartphones and tablets run apps that provide broader functionalities beyond their core features [10]. Owing to these advancements, smartphones and tablets have been used differently, most notably, through the collection of patient data with an array of sensors, the use of decision-making algorithms, and the presentation of visually augmented and interactive longitudinal data [11,12]. Such vastly different utility warrants a distinction between the traditional mHealth interventions based on cellular phones and modern smart device-based interventions. We define *smart device-based mHealth interventions* as mHealth interventions that use a smartphone, tablet, or peripheral device and their unique features, including high computing power, advanced functions using apps, use of sensors, fast network speed, and the interactive multitouch screen, to deliver health care services.

Smart devices have inherited the characteristics of cellular phones, and they are portable, pervasive, and becoming more and more affordable. In early 2016, the adoption rates of smartphones and tablets by older adults aged 60 to 69 years were 46% and 41%, respectively [13]. Older adults also have expanded the way they use smartphones and tablets, with health information seeking being the second most frequently executed task besides making phone calls [14]. With the growing older population, it is expected that more and more older adults will be interested in incorporating these smart devices into their chronic disease management.

As smart devices started to be widely adopted in the early 2010s, a reflective increase in mHealth research activities beginning in 2010 was noted in previous studies [11,12,15]. The randomized controlled trial (RCT) was identified as the most common research design, followed by descriptive and feasibility studies [11]. One study reported texting (short message service, SMS) as the most frequently used intervention tool, followed by apps on smartphones, noting its significant and growing role [11]. Two systematic studies reported overall positive patient outcomes from mHealth interventions but did not specify which clinical measures had the most benefit [11,15]. Another scoping review examined the design, development, and evaluation of mHealth systems for community-living older adults [12]. This study identified significant strategies for successful adoption among older adults, including user-centered design, collaborative team approaches, change management tactics for improving organizational and systems readiness, and formative and summative evaluation for improved user interfaces [12]. However, this study did not assess the characteristics and nature of smart device-based interventions and their impact on patient outcome. Overall, previous research had a vague distinction between the traditional and smart device-based interventions and lacked focus on the geriatric population.

To accommodate the broader functionalities and wide adoption of smart devices that can potentially better support chronic disease management for older adults, there is a need to synthesize research activities and evidence regarding smart device-based interventions. This scoping review explored this knowledge gap by mapping the extent and nature of the available literature, and will provide future directions for smart device-based interventions for older adults managing chronic disease.

## Methods

### Study Design

We chose to conduct a scoping review because the topic of smart device-based mHealth has emerged across a wide range of disciplines and it includes diverse chronic conditions. In particular, we used the Arksey and O’Malley framework for a scoping review [16] and the revisions made by Levac et al [17]. We also used thematic synthesis of qualitative studies to contextualize the findings of the quantitative studies.

### Framework Stage 1: Identifying the Research Question

The first stage began with the previously described literature review, followed by the identification of the knowledge gap. To comprehensively map the extent and nature of smart device-based mHealth research activities for chronic disease management for older adults, this scoping review set out to answer 4 research objectives: (1) to understand the range and methods of biometric measurements and patient-reported outcome measures (PROMs) in smart device-based mHealth interventions, (2) to describe the types of self-management support employed in smart device-based mHealth interventions, (3) to describe the impact of smart device-based mHealth interventions on clinical outcomes, and (4) to thematically synthesize older adults’ experience with smart device-based mHealth systems.

### Framework Stage 2: Identifying Relevant Studies

We searched 4 databases—MEDLINE, Embase, CINAHL, and Web of Science—using search terms related to smart devices, older adults, and chronic diseases. For smart device-related studies, we searched for smartphones, tablets, wearable devices, and fitness trackers. For chronic diseases, we searched for type 1 diabetes mellitus and type 2 (T2DM) diabetes mellitus, cardiovascular diseases, chronic lung diseases, cancer, arthritis, and the catchall term chronic disease. We searched for cardiovascular diseases including hypertension, coronary artery disease, heart failure, and stroke. Chronic lung diseases included asthma and chronic obstructive pulmonary disease (COPD). We used appropriate subject headings (eg, Medical Subject Headings) when possible (Multimedia Appendix 1 shows the search strategies). Lastly, we reviewed the bibliographies of included documents to identify additional studies.

### Framework Stage 3: Selecting the Studies

To align the selected studies to the purpose of the scoping review, we used an iterative process of developing inclusion and exclusion criteria. Inclusion criteria were smart device-based mHealth studies that (1) explicitly recruited older adults, or where the average age of participants was 50 years or older, (2) aimed to support chronic disease management, (3) were published in 2010 or after, and (4) were written in English. Only articles published in 2010 or after were selected to accommodate the introduction of tablets and the wide adoption of smartphones [18]. The exclusion criteria were (1) SMS or interactive voice response-based mHealth interventions, (2) studies that validated electronic versions of scales or questionnaire forms of existing instruments, (3) smart device-based interventions for postoperative monitoring, and (4) studies that described the design, development, or usability evaluation of smartphone-based mHealth systems. A single reviewer screened for the title and abstract first, followed by a full-text review.

### Framework Stage 4: Charting the Data

This stage adopted the Arksey and O’Malley framework, an iterative process of creating and revising a data extraction form to add or remove variables as the author’s knowledge on the topic increased [16]. To optimize the data extraction and charting process, we created an Excel file (Excel 2011 for Mac; Microsoft Corporation) to collate the following variables that were pertinent to the aims of this research: (1) bibliographies, (2) chronic condition, (3) study design, (4) biometrics and PROMs, (5) type of self-management, (6) type of decision support, and (7) clinical and health outcomes.

### Framework Stage 5: Collating, Summarizing, and Reporting the Results

The 3 substages of this stage were (1) analyzing the data, (2) reporting results, and (3) interpreting the results. For the first substage, we assessed the basic numerical analyses of the extent and distribution of the studies. The Arksey and O’Malley framework recommends adopting a theoretical framework to collate and summarize the extracted variables in a systematic manner [17]. Based on the extracted variables resulting from framework stage 4, we uncovered a high resemblance to the 3 elements of the chronic care model (CCM) [19,20] (Table 1 [19-22]). We borrowed these elements from the CCM as analytical themes to report and interpret the results. The CCM is a widely accepted framework that primarily aims to improve overall chronic care [23]. The selected 3 elements of the CCM recognize the significance of clinical information systems and technologies to better support chronic disease, thus aligning well with the purpose of this study [20]. Using a portion of the CCM allows for a structured approach to reporting results, and thereby enhances the articulation of the results to readers [16].

We could not organize results from the qualitative and mixed-methods studies within the descriptive numerical summaries due to the incommensurable nature of these two data types. Instead, we conducted thematic synthesis to capture and synthesize qualitative data of the patient experience, following the protocol outlined by Thomas and Harden [24]. Line-by-line coding, construction of descriptive themes, and development of analytical themes were completed using EPPI-Reviewer 4.0 [25].

Studies were then grouped and reported into the following categories to answer the specific research objectives from framework stage 1: (1) study characteristics, (2) the 3 elements of the CCM, (3) clinical and health impact, and (4) patient perspectives.

**Table 1:** Mapping and rationale for mapping to the charted variables of the chronic care model (CCM).

Extracted variables	CCM	Description	Relevance to the extracted variable
Biometrics and patient-reported outcome measures	Clinical information systems	This element in the original CCM pertains to the use of patient, care, and outcome information to gain feedback, improve practice, and develop shared care plans [20]. This idea within the context of eHealth has evolved to accommodate advanced information technologies such as electronic medical records, personal health records, smartphones, and wearable devices. [21]	This extracted variable is the information collected from smart devices. The CCM claims that the role of clinical information systems is to capture information about patients, care, and outcomes to support medical practices. Biometrics and patient-reported outcome measures are often captured and remotely sent to clinicians to support clinical practice.
Type of self-management	Self-management support	The CCM puts patients at the center of chronic disease management, and they are considered the principal caregiver. Patient education to teach necessary skills, tools to monitor symptoms, and routine assessment are integral components of self-management support [22].	Various self-management techniques that were employed by participants were extracted and categorized. These self-management techniques correspond to the acquired skills, use of tools, and assessment of accomplishments for chronic disease management.
Type of decision support	Decision support	Decision support refers to the integration of evidence-based clinical guidelines, protocols, and standards for health care providers.	This variable was extracted to identify various methods of communicating with patients to support clinical decision making.
Not applicable	Delivery system design	Delivery system design is an element that draws on the restructuring of medical practices with clearly redefined roles to support patients with chronic diseases.	No study that met the inclusion criteria directly examined the impact of altered medical practice. However, the lack of redesign of delivery systems and its impact on workload is captured in the qualitative analyses.
Not applicable	Health care organization	Health care organization is the foundational component for the model. It mainly revolves around the alignment of goals and visions across the health care organizational structure, and policies on care delivery, reimbursement, and patients.	The scope of this review study focused at the level of individual interventions rather than policy and organizational development. Thus, the nonincluded studies examined health care organization. An evaluation study may be more appropriate to assess the relationship between health care organizational change and its impact on chronic disease care for individual patients.
Not applicable	Community resources and policies	Community resources and policies refer to the need for sufficient resources within community settings (eg, exercise programs), so that primary clinics can link patients to adequate support for chronic disease management.	Smart device interventions are still at the early stages of development. The availability of community resources to support these interventions is not found within the studies.

## Results

### Search and Screening Results

**Figure 1:**
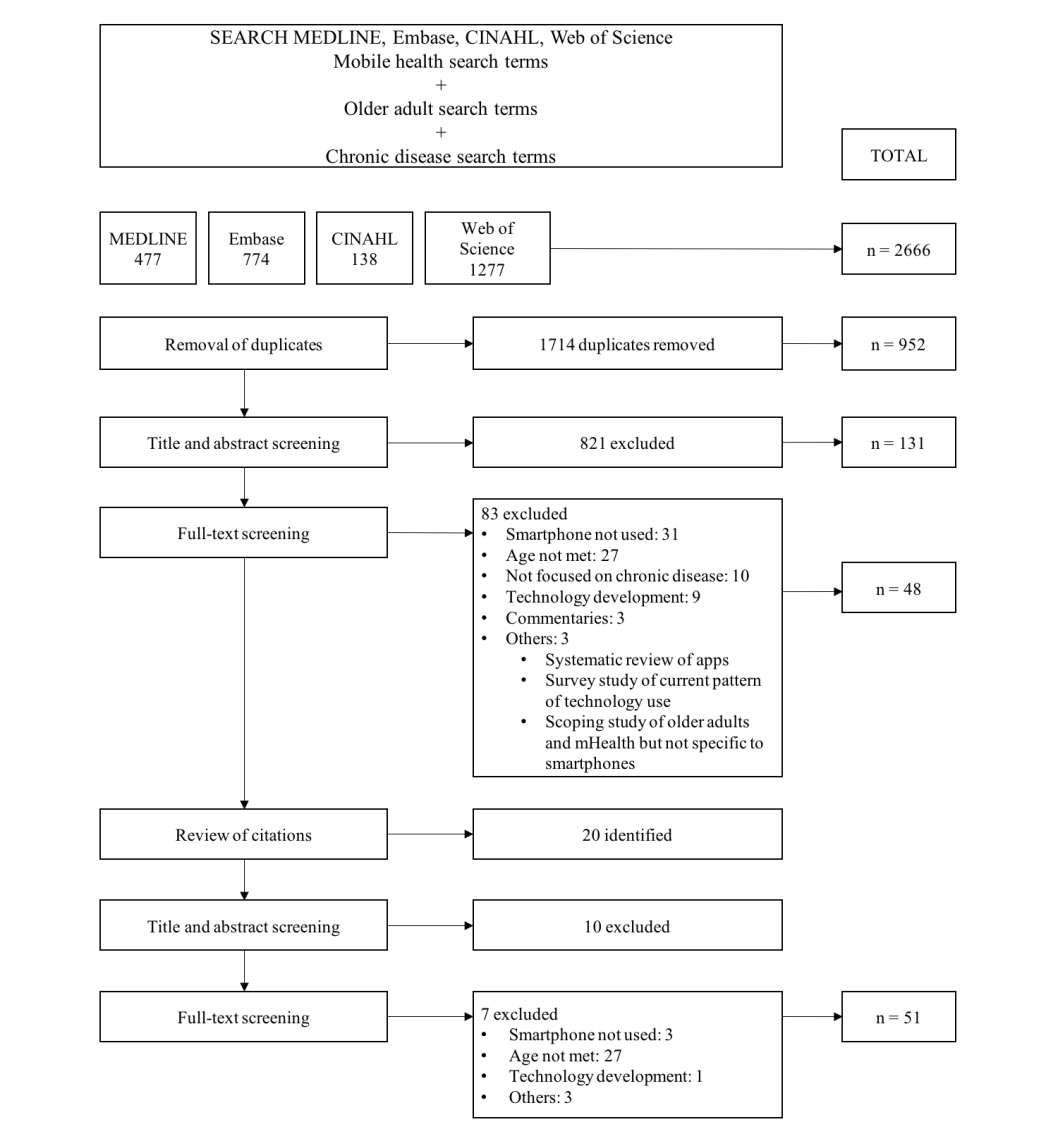
Flowchart of the study selection process and the reasons for exclusion.

The search of the 4 online databases in April 2016 yielded 2666 articles. We exported them to Mendeley reference management software (Mendeley for Mac version 1.17; Mendeley Ltd), where 1714 duplicates were removed. Title and abstract screening resulted in the exclusion of 821 articles, and we reviewed the remaining 131 articles in full. In total, we selected 48 articles and through review of the bibliographies identified an additional 10 potentially relevant articles. Repeating the screening cycle yielded an additional 3 articles. Figure 1 charts the detailed search, screening, and exclusion results. Ultimately, we included 51 articles (Multimedia Appendix 2 [26-76]), of which 44 were journal articles, 6 were poster abstracts, and 1 was from a conference proceedings. One poster abstract [26] and a journal article [27] described the same study.

**Figure 2:**
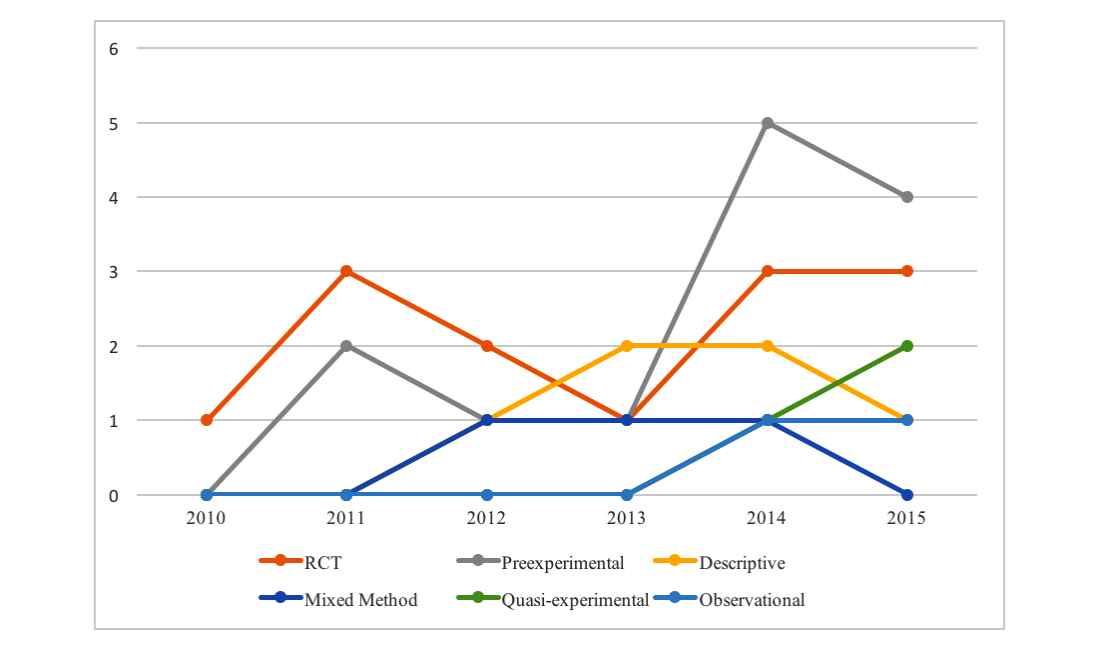
Type and number of study designs over time from 2010 to 2015. RCT: randomized controlled trial.

### Descriptive and Numerical Summary

The sources of the studies were mainly journal articles (43/51, 84%), conference poster abstracts (6/51, 12%), and conference proceedings (2/51, 3%). The study designs varied vastly. Preexperimental design studies (16/50, 32%) that included pilot and feasibility studies employing a single-group, pretest, and posttest design were most common. We also found RCTs (13/50, 26%), qualitative studies (6/50, 12%), quasi-experimental studies (5/50, 10%), and mixed methods (4/50, 8%). Study sample size ranged from 4 to 471, and intervention duration lasted from a few hours for interviews and focus groups to 1 year. Overall, there was a pattern of increasing smart device-based research activities over time, with a noticeable spike in 2014. Figure 2 presents the yearly pattern and the different types of study designs between 2010 and 2015. The figure excludes 2016, as we included only the first 4 months, secondary data analyses (3/50, 6%), and a study protocol (1/50, 2%), as they were not primary research studies.

Of the 46 primary studies (excluding the protocol, n=1; secondary data analysis studies, n=3; and poster abstract, n=1), 16 focused on T2DM (35%), 9 focused on chronic heart failure (20%), 5 focused on COPD (11%), 4 focused on hypertension (9%), 3 focused on cancer (7%), 1 focused on asthma (2%), and 1 focused on rheumatoid arthritis (2%); 7 studies examined more than 1 chronic condition in a single study (15%) and all examined T2DM management as a major topic.

### Chronic Care Model

A total of 3 extracted variables from each article were congruent with 3 of 6 elements in the CCM. The 3 extracted variables were biometrics and PROMs, type of self-management support, and type of decision support (Table 1).

#### Clinical Information Systems: Smart Devices Collecting Biometrics and PROMs

The smart devices were used for the collection of biometrics and PROMs—patient-measured information regarding their health status—and for the exchange of these data between health care providers and patients [77]. These tasks seamlessly align with 1 element of the CCM: clinical information systems. To explore the effective use of the smart devices as a clinical information system, this study examined the heterogeneity of the biometric data collected, and the methods of data generation and transmission.

**Table 2:** Number of studies collecting biometric measurements for each chronic disease.

Measurements	T2DM^a^	Heart failure	Multimorbidities	COPD^b^	Hypertension	Asthma	Total
Blood glucose	14		2				16
Body weight	4	6	2	1			13
Blood pressure	4	4	3		1		12
Step count	6		3	1			10
Heart rate		3		2	1		6
Oxygen level		1		2			3
Temperature				1			1
Peak expiratory flow rate						1	1
Electrocardiogram		1					1
Total	28	15	10	7	2	1	63

^a^T2DM: type 2 diabetes mellitus.

^b^COPD: chronic obstructive pulmonary disease.

**Table 3:** Number of studies collecting patient-reported outcome measures (PROMs) for each chronic disease.

PROMs	T2DM^a^	Hypertension	Cancer	Multimorbidities	Heart failure	Rheumatoid arthritis	COPD^b^	Asthma	Total
Symptoms	3	1	3		3			1	11
Medication adherence	2	2		2	1	1			8
Diet	6								6
Exercise	4			1					5
Well-being		1		1		1	1		4
Photos	2		1						3
Gait						1			1
Total	17	4	4	4	4	3	1	1	38

^a^T2DM: type 2 diabetes mellitus.

^b^COPD: chronic obstructive pulmonary disease.

A total of 40 studies used smartphones and peripheral devices to measure biometrics and PROMs [28,29,31-37,39-54, 56,57,59-61,63,65,66,68-72,74,76]. We identified heterogeneity in the collection of biometrics measurements (Table 2) and PROMs (Table 3) for each chronic disease between and within chronic diseases.

Data transfer from the peripheral devices and the hosting smartphone or tablet were either manual, relying on patients to record the data on the smartphone, or automatic, via Bluetooth, near-field communication, or universal serial bus. Manual recording of the measured data created an additional burden on patients [51]. The added burden potentially influenced overall compliance with the intervention, thus altering the overall impact on chronic disease management [57]. The degree of automation of data transfer was only 62% (39/63) for biometric measurement despite technical capabilities for automation (Table 4).

#### Self-Management Support: Strategies for Supporting Self-Management

Another element of the CCM is self-management support. The extracted variable “types of self-management” fits well with this CCM element, as we charted the studies to understand diverse self-management support strategies that were embedded within smart device-based interventions.

We identified 8 strategies to support self-management of chronic disease through smart devices (Textbox 1) among 40 articles [27-29,31,34-37,39-54,56,57,59-61,63,65,66,68-72,74-76]. Each strategy was distinct, yet they were often highly associated with one another. For example, automated feedback sent to patients immediately after taking measurements contains information that may overlap with patient education.

**Table 4:** Degree of automation of biometric measurements.

Measurements	Data transfer method, n (%)		Total
	Automatic	Manual	
Blood glucose	11 (69)	5 (31)	16
Blood pressure	7 (58)	5 (42)	12
Body weight	6 (46)	7 (54)	13
Electrocardiogram	1 (100)	0 (0)	1
Oxygen saturation %	2 (67)	1 (33)	3
Peak expiratory flow rate	0 (0)	1 (100)	1
Pulse	3 (50)	3 (50)	6
Step count	9 (90)	1 (10)	10
Temperature	0 (0)	1 (100)	1
Total	39 (62)	24 (38)	63

**The 8 identified self-management strategies.**

1. Self-monitoring: self-monitoring of the various biometrics, symptoms, medication, or healthy behaviors.
2. Patient education: education of patients pertinent to disease outcomes, self-monitoring, interpretation of measurements, benefits and risks of healthy behaviors, and medication and side effects.
3. Reminders: reminders for medication, self-monitoring, or behavior change.
4. Automated feedback: feedback content including motivational messages, educational messages, or how patients’ values compare with a clinical guideline.
5. Coaching: active coaching involving structured and predefined sessions with health care providers through in-person, over-the-telephone, and virtual interactions for the purposes of education, motivation, and discussion about self-management strategies.
6. Goal setting: individualized goal setting for the treatment or behavior change.
7. Treatment plan: treatment plan outlining a protocol to follow when patients experience exacerbations of symptoms.
8. Social support: sharing of the self-management progress to engage family members and friends.

Overall, there was an inverse relationship between the number of studies and the number of self-management support strategies employed. A total of 14 studies (35%) used a single self-management support strategy and 11 studies (28%) used 3 strategies; 8 (20%), 4 (10%), and 3 (8%) articles incorporated 3, 4, and 5 types of self-management support, respectively.

The most frequently used self-management technique was self-monitoring, found in all but 2 articles [27,75] (Table 5). The second most frequently used strategy was automated feedback [37,43,48,49,51,54,56,59,63,66,68-71,76]. The nature and extent of the automated feedback varied between studies. The most frequent form of automated feedback (9/15, 60%) related to the collected measurements (eg, blood pressure) or patient progress (eg, number of steps) against the predefined parameters or goals [43,48,51,59,66,68-71]. Other feedback included motivational, self-care, and educational messages. Patient education was another frequently used type of self-management support, and the extent of patient education differed considerably. Of the 13 smart device systems, 9 had a dedicated user interface for patient education; 7 of these included videos and audio files on topics ranging from instructions on proper use of peripheral devices, to general information about the disease, to proper exercise techniques [27,36,39,43,51,65,75]. Two studies merely provided a brief educational session before the start of the research study and supplemented the session with pamphlets and information sheets [47,66]. One study that aimed to enhance medication adherence provided information about each medication the patient was taking [52].

**Table 5:** Frequency of self-management support strategies employed within 40 studies.

Types of self-management support	Number of studies
Self-monitoring	38
Automated feedback	15
Patient education	13
Reminder	8
Coaching	8
Goal setting	6
Social support	2
Treatment plan	1

#### Decision Support

The CCM describes decision support as a function of the advanced clinical information system. The purpose of a decision support system (DSS) is to assist health care providers to adhere to an evidence-based clinical guideline. Within the context of smart device-based interventions, the support was provided directly to patients rather than through health care providers. Patients received support for decision making about self-management from diverse sources, categorized based on the medium of delivery as (1) virtual (a form of online communication, usually messaging), (2) in person, (3) by telephone, or (4) through a DSS.

A total of 23 articles documented aid in the decision-making process for patients. Of these, 14 studies (61%) integrated 1 type of decision support mechanism [28,34,37,39,42,47, 54,63,65,66,68,70,72,76] and 8 studies (35%) integrated more than 1 type of decision support mechanism [36,41,43,45, 48,56,59,69]. These interventions were designed to delay the involvement of health care providers to reduce the burden of monitoring patients. In total, 12 studies (52%) integrated a DSS [37,41,43,47-49,54,56,59,63,69,70]. The comprehensiveness and setup of the DSSs varied considerably: 3 smart device system-implemented DSSs not only determined the severity of the disease, but also recommended adjusting medications or starting a medication regimen based on the patient’s biometrics and PROMs [43,63,70]. Of 4 DSSs operating on evidence-based guidelines [37,43,47,70], 3 recommended medication adjustments. Another 4 studies allowed health care providers to determine the individualized parameters for each patient, and the DSS generated alerts based on these predefined values [48,49,59,69].

### Clinical and Health Impact

We assessed the impact of smart device-based interventions on patients’ clinical and health outcomes. Since this was not a systematic review, we did not assess the quality of the evidence; rather the review was aggregated and observational.

Of the 51 studies, 10 RCTs and 2 quasi-experimental studies examined clinical outcomes [36,42,45,47,48,50,56,59,63,69, 71,76]. The degree of clinical impact varied for different conditions and each clinical measure (Table 6).

The most frequently reported health improvement was HbA_1c_ control among patients with diabetes, while no superior clinical impact on blood lipid profile was observed [36,42,45,48,56,71]. Patients with asthma showed improvements in the percentage predicted peak expiratory flow rate and forced expiratory volume in the first second of expiration, but these findings were based on 1 RCT [47]. Patients with gastric cancer who had undergone gastrectomy and used smart devices to manage symptoms and nutritional intake were able to retain their body weight significantly better than the control group [76]. No clinical improvement was reported for heart failure and COPD intervention studies [59,62,69].

### Impact on Quality of Life

Many smart device-based chronic disease management programs aimed to manage symptoms and improve quality of life rather than solely focusing on improving clinical outcomes. Quality of life was assessed in 9 studies, and 6 assessment instruments were used [28,37,42,47,49,54,59,66,68]. Only the 36-Item Short Form Health Survey (SF-36) and the EuroQoL Five Dimensions Questionnaire (EQ-5D) were each used in 2 studies (Table 7).

A total of 3 RCTs reported significant improvements in the quality of life for patients with heart failure and asthma [37,47,59]. An RCT for patients with diabetes and COPD documented a mixed result of improvements in the physical component, but not in the mental component [66]. Another diabetes management intervention also reported a mixed result on quality of life. Patients reported less physical pain by the end of 4 weeks but no improvements in other domains of the SF-36 [54].

**Table 6:** Number of clinical outcomes of randomized controlled trials and quasi-experimental design studies of smart device-based mHealth interventions.

Chronic diseases (measurements)	Significant outcomes	Nonsignificant outcomes
T2DM^a^ (hemoglobin A_1c_)	3	1
T2DM (blood pressure)	1	4
T2DM (lipids)	0	5
HF^b^ (brain natriuretic peptide, LVEF^c^)	0	2
HF (self-care activities)	1	1
HF (lipids, blood pressure, weight, waist circumference)	0	1
Chronic obstructive pulmonary disease (dyspnea, fatigue level)	0	1
Asthma (peak expiratory flow rate, FEV_1_ % pred^d^)	1	0
Cancer (body weight)	1	0
Hypertension (blood pressures)	1	0
Total	8	15

^a^T2DM: type 2 diabetes mellitus.

^b^HF: heart failure.

^c^LVEF: left ventricular ejection fraction.

^d^FEV_1_ % pred: percentage predicted forced expiratory volume in the first second of expiration.

**Table 7:** Smart device-based mHealth interventions and impact on quality of life.

Study (first author, date, reference no.)	Study design	Morbidity	Impact on quality of life	Measurement tool
Anglada-Martinez, 2016 [28]	Preexperimental	Multimorbidities	Not significant	EQ-5D^a^
Hägglund, 2015 [37]	RCT^b^	Heart failure	Significant	KCCQ^c^
Karhula, 2015 [42]	RCT	T2DM^d^ and heart failure	Not significant	SF-36^e^
Liu, 2011 [47]	RCT	Asthma	Significant	SF-12^f^
Maguire, 2015 [49]	Preexperimental	Cancer	Not significant	FACT-L^g^
Quinn, 2015 [54]	Preexperimental	T2DM	Mixed	SF-36
Seto, 2012 [59]	RCT	Heart failure	Significant	MLHFQ^h^
van der Weegen, 2015 [66]	RCT	T2DM and COPD^i^	Mixed	RAND-36^j^
Verwey, 2014 [68]	Mixed methods	COPD	Significant	EQ-5D

^a^EQ-5D: EuroQoL Five Dimensions Questionnaire.

^b^RCT: randomized controlled trial.

^c^KCCQ: Kansas City Cardiomyopathy Questionnaire.

^d^T2DM: type 2 diabetes mellitus.

^e^SF-36: 36-Item Short Form Health Survey.

^f^SF-12: 12-Item Short Form Health Survey.

^g^FACT-L: Functional Assessment of Cancer Therapy – Lung.

^h^MLHFQ: Minnesota Living with Heart Failure Questionnaire.

^i^COPD: chronic obstructive pulmonary disease.

^j^RAND-36: RAND 36-Item Health Survey.

**Table 8:** Descriptive and analytical themes identified in 5 smart device-based mHealth research studies.

Themes		Hallberg, 2016 [38]	Maguire, 2015 [49]	Nes, 2012 [51]	Verwey, 2014 [68]	Williams, 2014 [73]
**Inappropriate target patients**						
	No symptoms and stable condition	Yes	N/A^a^	N/A	Yes	N/A
**Insufficient training**						
	Insufficient knowledge on how to use the system	Yes	Yes	N/A	Yes	N/A
	Insufficient knowledge on how to interpret data	Yes	N/A	Yes	N/A	Yes
	Anxiety toward technology use	N/A	N/A	N/A	N/A	Yes
**Recognition of benefits**						
	Increased self-awareness of symptoms and disease conditions	Yes	Yes	Yes	Yes	Yes
	Increased motivation to upkeep chronic disease management	Yes	Yes	Yes	Yes	Yes
	Increased knowledge about chronic disease and management	Yes	N/A	N/A	N/A	Yes
	Increased involvement and engagement in chronic disease management	Yes	N/A	Yes	N/A	Yes
	Improved chronic disease management behaviors and outcomes	N/A	Yes	Yes	N/A	Yes
	Utility as a communication tool	Yes	Yes	N/A	N/A	Yes
**A sense of connectedness and reassurance**						
	Feeling assured	N/A	Yes	N/A	N/A	Yes
	Reduced uncertainty around chronic disease management	N/A	Yes	N/A	N/A	Yes
	Personalized feedback, advice, and messages	N/A	Yes	Yes	Yes	N/A
**System issues diminish motivation**						
	Usability issues	Yes	N/A	Yes	Yes	N/A
	Perceived ease of use	Yes	Yes	N/A	N/A	Yes
	Feeling burdened	N/A	N/A	Yes	N/A	Yes

^a^N/A: not applicable.

### Thematic Synthesis

For the thematic analysis, we chose 5 smart device-based mHealth research studies that aimed to explore patient perspectives [38,49,51,68,73]. Initially, we constructed 16 descriptive themes from line-by-line coding. We condensed the descriptive themes into 5 analytical themes (Table 8), then further categorized them as the facilitators and barriers to successful adoption by older adults.

#### Facilitators

##### Theme 1: A Sense of Connectedness and Reassurance

Patients understood that their measurements and recordings were observed by health care providers. A sense of reassurance was offered when patients received feedback from clinicians.

...the rapid feedback by health professionals in response to reported symptoms...it just keeps your morale up...(pg E43).

49

A sense of connectedness and feeling of reassurance was offered regardless of whether health care providers were monitoring the system or not.

Yet the virtual link offered by mHealth intervention appeared to reassure patients and gave a sense of continuity of care...the sharing of patients’ self-monitoring data with the research nurse, even though this was infrequent and did not replace current care (pg e395).

73

Having the option to contact health care providers at any time reduced uncertainties that arose when self-managing symptoms and evoked a sense of reassurance.

...reduced uncertainty experienced by the patients, particularly at times when they were at home and were unsure as to whether they should contact health professionals or not (pg E43).

49

##### Theme 2: Recognition of the Benefits of Using Smart Devices

Many participants from multiple studies commented on the increased awareness, motivation, and engagement in chronic disease management owing to the use of smart device systems. Patients frequently reflected on their self-management behaviors while filling out diaries for PROMs, noting it enhanced their sensitivity to and awareness of their symptoms.

...filling in the diaries gave them better insight into their diabetes, increased their coping and self-management strategies...increasing their honesty in answering the diary questions (pg 389-90).

51

...reviewing their condition and how they felt not just over a number of days but also within a 24-hour period...As patients were answering questions about their symptoms as well as monitoring their oxygen saturation on daily basis, they felt encouraged to think more about how they were feeling each day and throughout the day (pg e395).

73

Patients were more motivated to comply with mHealth interventions by achieving predefined goals. Also, patients recognized the significance of complying with an mHealth program when they experienced the relationship between their self-management behaviors and its impact on their health.

They felt encouraged to be more active and mentioned three aspects [that were positive about the intervention]: the awareness of their physical activity performance, the stimulating effect of the daily target goal and the positive effect on self-efficacy (pg 31).

68

This became evident to those who missed their antihypertensive medication at some point and then personally detected that their blood pressure had gone up the same day (pg 144).

38

#### Barriers

##### Theme 3: Incompetent Recruitment Strategy

Smart device-based systems did not offer much value for some recruited participants, although this varied given patients’ chronic disease management status. These patients consistently commented on the lack of value the intervention added to their management strategies. In one study that targeted hypertensive patients, the authors described the experience of those who were noncompliant with the mHealth program:

...patients who did not perceive any symptoms or who had stable blood pressure found some questions to be less relevant (pg 143).

38

Another study that promoted physical activity among patients with COPD identified the reasons for lower ratings when asked if the intervention had a positive effect on their physical activity level.

A total of 12 patients were positive about the effect of the intervention on their physical activity performance and five patients were neutral about it; the latter were patients who were already sufficiently active (pg 31).

68

Smart device systems that visually represented patients’ longitudinal progress were viewed as less useful, since patients with stable conditions were well aware without seeing the graph.

...they did not consider it necessary to look at the graphs, as their answers and values were quite similar without significant variation (pg 143).

38

Although many studies had explicit eligibility criteria to recruit appropriate patients, they did not filter out patients who were already sufficiently managing chronic diseases. Alternatively, the targeting of unstable patients for smartphone-based mHealth interventions could pose a threat to their safety.

##### Theme 4: Insufficient Training

The theme of insufficient training encompasses 2 distinct issues. First, it refers to a lack of training and follow-up assistance for using smart device systems.

Some of the participants commented on how they were never trained on using this component of the system (pg E43).

49

Moreover, some smart device systems were set up to operate with computers, expanding the need for technical training. Participants did not pursue extra help and often did not use a portion of, or an entire, system. This was evident in a study where patients had an option to view their progress on a computer.

...simply logging into the computer to look at the graph was a problem in itself because they had forgotten how to do this. Therefore, some patients did not in fact look at the graphs themselves in their own home...(pg 143).

38

Another study highlighted the need for additional training, noting that not all participants learned to use the system at the same pace.

A total of six patients needed some extra advice about how to log in, which was given to them (pg 32).

68

Second, there was a lack of patient education to support the self-management and interpretation of measured biometrics.

The participants had doubts especially about how to interpret these questions [referring to questions for daily diaries (pg 390).

51

...patients were uncertain how to interpret oxygen levels (pg e396).

73

The lack of knowledge on how to interpret questions and the measured biometrics and PROMs led to inaccurate reporting of their symptoms or left participants uncertain about their conditions and progress. Insufficient and inadequate levels of training had a leaching effect on nurses and therapists, with an unanticipated increase in workload due to issues such as longer consultations [51,68].

##### Theme 5: Diminished Motivation Due to Usability and Technical Issues

Usability and technical issues were prevalent across the studies, and they led to frustration among older adults and had a detrimental effect on patient motivation to continue with the study.

Although most of the patients were positive about the tool, the motivation of some patients dropped when technical problems occurred (pg 32).

68

Participants reported technical problems as frustrating and demotivating (pg 391).

51

Most studies reported varying degrees of technical and usability issues with smart systems. These issues are unavoidable in any technology-driven intervention, but no study described a risk mitigation strategy in case of technology failure. Patients, more and more, play a critical role in managing lifelong illnesses, and smart device-based systems can play a central role in delivering necessary support. As a result, these issues posed a significant threat to effective use of the tool, thus hindering chronic disease management for some older adults.

## Discussion

### Limitations of Smart Device-Based Research

This scoping review explored the extent of the use of smartphones, tablets, and peripheral devices, the diverse self-management support strategies, and the nature of decision support and DSSs for managing chronic diseases among older adults. Further, this study illustrated older patients’ experience with using such technologies for their care.

#### Lack of Gerontological mHealth Research

Overall, smart device-based research for older adults began to rise with a noticeable increase in 2014. This is similar to previous literature findings [11]. Despite the increased research activities, only 3 studies reviewed here explicitly targeted older adults, revealing a lack of gerontological smart device-based studies [50,54,74]. This is a lost opportunity as more and more older adults are displaying interest in using smartphones and tablets for obtaining health information [78]. This trend is expected to grow as the proportion of older adults living with chronic conditions is increasing.

#### Dominance of Preexperimental Study Design

mHealth studies have long been criticized for the dominance of preexperimental studies that labeled themselves as pilot, feasibility, and field-test studies [5,12], and this trend continued among smart device-based mHealth research. These types of studies lacked a control group and are subject to threats to internal validity [79]. Consequently, these studies offer limited knowledge about the effectiveness of smart device-based interventions in producing meaningful clinical outcomes compared with RCTs and quasi-experimental studies [5]. However, a strong dominance of pilot studies and an increasing number of RCTs is indicative of overall progress in this topic.

#### Narrow mHealth Research Scope

Diabetes and hypertension management has been the dominant subject for traditional mHealth research, whereas other chronic disease management received very limited attention [2,4]. This pattern strongly continued among smart device-based mHealth research. More chronic diseases have been explored using smart devices, including heart failure, cancer, asthma, and multichronic disease management. This expansion of the scope of mHealth research is a welcome change, and these studies have provided deeper understanding of the potential for smart devices in chronic disease management for older adults. However, the scope of mHealth research should expand further as technological capabilities of smart devices increase, but it was too early for this scoping review to detect meaningful diversification.

#### Inadequate Support for Participants

The literature provides very limited information about the nature of training (ie, number of participants per training session; number of hours) and the content of the training (ie, how to use a smartphone; how to interpret blood glucose levels). However, the thematic synthesis of qualitative studies unveiled an issue with research practice in regard to training of participants. Moreover, there was no consistent structure for ongoing support for participants. No standard practice for participant training on technology-driven interventions had many participants discouraged from using smart devices to their full potential and often led to frustration among health care providers.

#### Insufficient Evidence for Health and Clinical Outcomes

The evidence for smart device-enhanced clinical and health outcomes was insufficient and mostly inconclusive. Smart device-based interventions for older patients with diabetes demonstrated its effectiveness in reducing the HbA_1c_ level, but it did not demonstrate superior clinical outcomes than the traditional mHealth interventions [80]. Similarly, minimal evidence was documented for improvement in chronic cardiovascular and chronic respiratory disease management. Many studies only reported recognizable clinical benefits among a particular group of patients through the use of subanalyses and emphasized the potential these new technologies hold for the future.

### Implications for Future Research

#### Need for Evidence-Based Design of Smart Device-Based Programs

We observed disjointed chronic disease care processes across studies, and even within the studies that focused on the same chronic conditions. Additionally, the lack of research activities that focused on the geriatric population is of great concern. The collection of a wide range of patient biometrics and PROMs, and the use of many different self-management strategies indicate the absence of standards for the evidence-based design of smart device-based programs for older adults.

Heterogeneity in intervention design is not unique to smart device-based interventions. Heterogeneity in the content of interventions for different chronic conditions, and even within a single chronic condition, was also reported in a scoping review that examined nontechnology-derived chronic disease self-management programs [81]. This study indicated that no single design would suffice for the design of smart device-based programs but, instead, the design of interventions should ensure that components of such programs are evidence based.

Research activities for older adults had a skewed focus on validating the effectiveness of a handful of components such as self-monitoring and automated feedback, which had been extensively researched through traditional mHealth interventions through the use of text messaging and self-monitoring devices [2,4,5]. These studies demonstrated limited clinical effectiveness for older adults for managing chronic diseases [2,4,5]. This finding is in line with behavioral change interventions for older adults in weight management and physical activity delivered via text messages and self-monitoring [2,82]. In contrast, mHealth intervention studies for younger populations in smoking cessation through text messaging demonstrated a more than doubled smoking cessation rate [2,4]. More importantly, diabetes management interventions via text messaging for adolescents and young adults demonstrated increased blood glucose monitoring frequency and clinically significant improvement in HbA_1c_ level [4]. Such a pattern indicates that smart device-based interventions for older adults need to exploit the technical advantages for testing and expanding new and novel strategies for self-management. Example strategies that used the new features of smart devices are social support through the use of social network services to involve others [83] and goal setting for successful behavior changes through visually presenting progress [83,84]. These are only a few examples of evidence-based strategies uncovered in this scoping review; future smart device-based interventions should explore other evidence-based self-management support strategies and validate their generalizability, thus leading to the development of more evidence-based chronic disease management programs.

#### Patient Decision Support Systems and Data-Driven Health Care

The role of DSSs within the context of smart device-based interventions is to support patients in their clinical decision-making needs, thereby increasing patient autonomy. Smart device-based interventions often delegate the responsibilities of clinical practices to patients with support from patient DSSs, but the degree of delegation varied widely. One study employed a DSS that initiated a medication regimen for patients based on their reported biometrics and PROMs [47]. Some studies developed simple rule-based DSSs that determined the health status of a patient automatically, and clinicians were only involved when a patient needed closer examination [43,48,49,56,59]. On the other hand, some DSSs were less intrusive by providing motivational messages, educational contents, and self-care advice [63,69].

Use of DSSs within smart device-based interventions raises multiple issues that warrant further research. First, the complete delegation of the clinical decision-making process to patients—albeit with validated DSSs—will need extensive research on its safety, efficacy, and implications for chronic care management and patient well-being. This may be an appropriate approach for simple situations where there is a single decision option with suitable patient education. However, many medical decisions will benefit from clinician involvement to allow careful examination of available decision options that can lead to informed shared decision making [85]. Some studies attempted to achieve this by setting up rules around when a clinician should be involved or by setting up regular scheduled supervision by clinicians. Too little interaction with clinicians or too much delegation of clinical responsibilities created uncertainty and anxiety among patients. Qualitative analyses demonstrated that online supervision was deemed sufficient to provoke the sense of connectedness (ie, online communication) to reassure patient confidence. The use of patient DSSs along with scheduled clinician supervision needs further research to optimize its impact on patient safety, patient autonomy, and resource utilization (ie, clinician time). Second, minimally intrusive DSSs that provide motivational messages and educational contents need further verification for their effectiveness in supporting self-management. Some research studies have investigated this topic, but the conclusions reported are insufficient, and the need for tailored messages was noted [38,62].

#### Expanding Practice Through the Use of Innovative Technologies

Overall, the adoption of innovative technologies was not well realized within smart device-based research. One aspect that was examined in detail was the diversity and the methods of gathering biometrics and PROMs. In particular, the automation of data generation and transmission was of significant importance for improving chronic disease care because it influenced older adults’ compliance with smart device-based interventions [34,42]. For example, the impact of automation was well documented in one study in which patients had to wear an accelerometer (automated) and fill in diaries every day (manual): the mHealth compliance rate was much higher with accelerometer use [63]. The effectiveness of automation in improving mHealth compliance was supported by other studies, especially for older adults [34,36]. However, over one-third of the biometrics were obtained through analog medical equipment that did not automatically transfer the data, and compliance was noted to be lower. Use of peripheral devices that can automate data generation and transmission is recommended for future smartphone-based research for older adults.

While the automation of data collection will benefit smart device-based mHealth research, we should look beyond this for more innovative solutions. The topic of wearable technologies for health care is emerging and receiving growing attention [86,87]. A recent industry report that scanned the consumer-grade wearable device adoption level indicated almost as high a level of adoption among older US adults as among their younger counterparts [88]. Wearable technologies have not been explored for chronic disease management for older adults. Continuous monitoring of many vital signs, along with the simplicity of devices, provides great potential for chronic disease management, especially for older adults [89]. Early research protocols and findings from studies that used smart wearable devices to support chronic disease management among older adults have begun to emerge, indicating that mHealth and ubiquitous health research practices are expanding [90,91].

Use of multiple sensors and continuous monitoring generate an enormous amount of data, which has opened up opportunities for advanced data analytics. Data analytics has demonstrated its effectiveness in medicine, from diagnostics, clinical decision support, and cardiac event detection [92-96]. Use of advanced data analytics has been integrated into mHealth studies, albeit very limited, for general health promotion and prevention research. One study used smartphones to generated personalized, contextualized, and actionable health advice and recommendations based on activity and nutrition intake [97]. Another study employed a neural network to predict falls based on kinematic parameters collected from a smart wearable device [98]. Future mHealth studies should explore the utility of data analytic techniques, potentially using large amount of data from continuous monitoring from smart wearable devices for detecting exacerbation among patients with COPD [99], irregular heart beat in cardiovascular disease [92], and dangerous spikes of blood glucose levels [100,101], to name a few, without resorting to clinicians. Ultimately, new innovative technologies should improve chronic disease care, increase the autonomy of patients, prolong the independence of older adults, and ensure overall well-being rather than being burdensome on patients’ daily lives.

### Study Limitations

This scoping study was subject to publication bias. Despite our effort to be as inclusive as possible by searching 4 separate reference databases, studies with negative findings may have been underrepresented. Another limitation of the study was the restrictive search of online reference databases and exclusion of gray literature. We excluded gray literature from this scoping review to balance the feasibility of this study with the available resources. This study was conducted by a single reviewer, which may have caused biases in selecting and screening of search results. Given the nature of the scoping review, this study did not synthesize evidence to determine the effectiveness of smart device-based tools. Instead, it captured the diversity of the available literature with its varied objectives, interventions, populations, and settings. Consequently, this study was more exploratory and suggestive of future research directions.

### Conclusions

Effective care of older adults with chronic conditions is a growing global priority. The advances in smartphones, wearables, and other smart devices align well with the developing interests of older adults to integrate technologies into their health care. Future smart device-based mHealth interventions should focus on implementing evidence-based strategies into the research design, exploring more powerful and reliable data-driven DSSs, and using innovative technologies to enhance and expand mHealth practice. To unlock the potential of rapidly advancing technologies for the aging population, future mHealth research should embrace innovation and look to develop evidence-based chronic care programs for older adults.

## Appendix

Multimedia Appendix 1 Final search queries for online reference databases.

Multimedia Appendix 2 Description of the 51 included articles.

## Abbreviations

CCM: chronic care model
COPD: chronic obstructive pulmonary disease
DSS: decision support system
EQ-5D: EuroQoL Five Dimensions Questionnaire
HbA1c: hemoglobin A1c
PROMs: patient-reported outcome measures
RCT: randomized controlled trial
SF-36: 36-Item Short Form Health Survey
SMS: short message service
T2DM: type 2 diabetes mellitus
