# Supplementary material for: Smart Devices for Older Adults Managing Chronic Disease: A Scoping Review
Source: JMIR Mhealth Uhealth. 2017 May 23;5(5):e69. doi: 10.2196/mhealth.7141 (PMC5461419; doi:10.2196/mhealth.7141)
Supplement: Multimedia Appendix 1 [file mhealth_v5i5e69_app1.pdf]

## Final search queries for online reference databases.

MEDLINE using the PubMed platform.

- ((mHealth*[TIAB] OR m-health*[TIAB] OR mobile-health*[TIAB] OR smartphone*[TIAB] OR iPhone*[TIAB] OR android[TIAB] OR blackberry[TIAB] OR cellphone*[TIAB] OR cell phone*[TIAB] OR cellular phone*[TIAB] OR mobile phone*[TIAB] OR mobile app*[TIAB] OR mobile device*[TIAB] OR mobile technolog*[TIAB] OR tablet computer*[TIAB] OR mobile tablet*[TIAB] OR electronic tablet*[TIAB] OR tablet PC[TIAB] OR iPad*[TIAB] OR iPod*[TIAB] OR wearable technolog*[TIAB] OR consumer wearable*[TIAB] OR (commercial*[TIAB] AND wearable*[TIAB]) OR fitness tracker[TIAB] OR activity tracker[TIAB] OR fitbit[TIAB] OR smart watch[TIAB] OR smartwatch[TIAB] OR cell phones[MeSH:noexp] OR mobile applications[MeSH] OR computer, handheld[MeSH] OR smartphone[MeSH]) AND (older adult*[TIAB] OR older patient*[TIAB] OR older person*[TIAB] OR older people*[TIAB] OR older user*[TIAB] OR older man[TIAB] OR older men[TIAB] OR older woman[TIAB] OR older women[TIAB] OR older individual*[TIAB] OR elder*[TIAB] OR senior*[TIAB] OR geriatric*[TIAB] OR aged[TIAB] OR old age[TIAB] OR aging[TIAB] OR ageing[TIAB] OR middle aged[MeSH] OR aged[MeSH]) AND (heart failure[TIAB] OR coronary artery disease*[TIAB] OR ischemic heart disease*[TIAB] OR coronary arterioscleros*[TIAB] OR coronary atheroscleros*[TIAB] OR COPD[TIAB] OR chronic obstructive pulmonary disease*[TIAB] OR chronic obstructive lung disease*[TIAB] OR chronic obstructive airway disease*[TIAB] OR asthma*[TIAB] OR diabt*[TIAB] OR hypertension[TIAB] OR high blood pressure[TIAB] OR stroke[TIAB] OR cancer[TIAB] OR

neoplasm*[TIAB] OR arthritis[TIAB] OR chronic disease*[TIAB] OR chronic illness*[TIAB] OR chronically ill[TIAB] OR heart failure[MeSH] OR pulmonary disease, chronic obstructive[MeSH] OR asthma[MeSH] OR diabetes mellitus[MeSH] OR hypertension[MeSH] OR stroke[MeSH] OR arthritis[MeSH] OR coronary artery disease[MeSH] OR neoplasms[MeSH] OR chronic disease[MeSH])

EMBASE using the Ovid platform.

- (mHealth*.mp OR m-Health*.mp OR mobile-health*.mp OR smartphone*.mp OR iPhone*.mp OR android.mp OR blackberry.mp OR cellphone*.mp OR cell phone*.mp OR cellular phone*.mp OR mobile phone*.mp OR mobile app*.mp OR mobile device*.mp OR mobile technolog*.mp OR tablet computer*.mp OR mobile tablet*.mp OR tablet computer*.mp OR mobile tablet*.mp OR electronic tablet*.mp OR tablet PC.mp OR iPad*.mp OR iPod*.mp OR wearable technolog*.mp OR consumer wearable*.mp OR (commercial*.mp AND wearable*.mp) OR fitness tracker.mp OR activity tracker.mp OR fitbit.mp OR smart watch*.mp OR smartwatch*.mp OR mobile application/ OR mobile phone/ OR smartphone/ OR (microcomputer/ AND (handheld*.mp OR handheld*.mp OR tablet*.mp))) AND (older adult*.mp OR older patient*.mp OR older person*.mp OR older people.mp OR older user*.mp OR older man.mp OR older men.mp OR older woman.mp OR older women.mp OR older individual*.mp OR elder*.mp OR senior*.mp OR geriatric*.mp OR middle age*.mp OR aged.mp OR old age.mp OR aging.mp OR ageing.mp OR aged/ OR very elderly/ OR geriatric patient/ OR middle aged/) AND (heart failure.mp OR coronary artery disease*.mp OR ischemic heart disease*.mp OR coronary arterioscleros*.mp OR

coronary atheroscleros*.mp OR COPD.mp OR chronic obstructive lung disease*.mp OR chronic obstructive pulmonary disease*.mp OR chronic obstructive airway disease*.mp OR asthma*.mp OR diabet*.mp OR hypertension.mp OR high blood pressure.mp OR stroke.mp OR cancer.mp OR neoplasm*.mp OR arthritis.mp OR chronic disease*.mp OR chronic illness*.mp OR chronically ill*.mp OR exp heart failure/ OR chronic obstructive lung disease/ OR exp asthma/ OR exp diabetes/ OR exp hypertension/ OR exp stroke/ OR exp arthritis/ OR exp coronary artery disease OR exp neoplasm/ OR chronic disease/) AND (LIMIT 4 TO (HUMAN AND ENGLISH LANGUAGE AND YR=”2010-CURRENT”))

CINAHL using the ESCO platform

1. (TI(mHealth* OR m-Health* OR mobile-health* OR smartphone* OR iPhone* OR android OR blackberry OR cellphone* OR cell phone* OR cellular phone* OR mobile phone* OR mobile app* OR mobile device* OR mobile technolog* OR tablet computer* OR mobile tablet* OR electronic tablet* OR tablet PC OR iPad* OR iPod* OR wearable technolog* OR consumer wearable* OR (commercial* AND wearable*) OR fitness tracker OR activity tracker OR fitbit OR smart watch* OR smartwatch*) OR AB(mHealth* OR m-Health* OR mobile-health* OR smartphone* OR iPhone* OR android OR blackberry OR cellphone* OR cell phone* OR cellular phone* OR mobile phone* OR mobile app* OR mobile device* OR mobile technolog* OR tablet computer* OR mobile tablet* OR electronic tablet* OR tablet PC OR iPad* OR iPod* OR wearable technolog* OR consumer wearable* OR (commercial* AND wearable*) OR fitness tracker OR activity tracker OR fitbit OR

smart watch* OR smartwatch*) OR MH(smartphone OR cellular phone OR mobile applications OR wearable sensors))

2. (TI(Older adult* OR older patient* OR older person* OR older people OR older user* OR older man OR older men OR older woman OR older women OR older individual* OR elder* OR senior* OR geriatric* OR aged OR old age OR aging OR ageing) OR AB(Older adult* OR older patient* OR older person* OR older people OR older user* OR older man OR older men OR older woman OR older women OR older individual* OR elder* OR senior* OR geriatric* OR aged OR old age OR aging OR ageing) OR MH(aged+ OR middle age OR young adult))
3. (TI(heart failure OR coronary artery disease* OR ischemic heart disease* OR coronary arterioscleros* OR coronary atheroscleros* OR COPD OR chronic obstructive lung disease* OR chronic obstructive pulmonary disease* OR Chronic obstructive airway disease* OR asthma* OR diabet* OR hypertension OR high blood pressure OR stroke OR cancer OR neoplasm* OR arthritis OR chronic disease* OR chronic illness* OR chronically ill) OR AB(heart failure OR coronary artery disease* OR ischemic heart disease* OR COPD OR chronic obstructive lung disease* OR chronic obstructive pulmonary disease* OR asthma* OR diabet* OR hypertension OR high blood pressure OR stroke OR cancer OR neoplasm* OR arthritis OR chronic disease* OR chronic illness* OR chronically ill) OR MH(heart failure+ OR pulmonary disease, chronic obstructive+ OR asthma+ OR diabetes mellitus+ OR hypertension+ OR stroke+ OR arthritis+ OR coronary disease+ OR neoplasms+ OR chronic disease))
4. 1 AND 2 AND 3

Web of Science

1. (TS=(mHealth* OR m-Health* OR smartphone* OR iPhone* OR android OR blackberry OR cell phone* OR cellphone* OR cellular phone* OR mobile phone* OR mobile app* OR mobile device* OR mobile technolg* OR tablet computer* OR mobile tablet* OR electronic tablet* OR tablet pc OR iPad* OR iPod* OR wearable technolog* OR consumer wearable* OR (commercial* AND wearable*) OR fitness tracker OR activity tracker OR fitbit OR smart watch* OR smartwatch*))
2. (TS=(heart failure OR Coronary artery disease* OR ischemic heart disease* OR coronary arterioscleros* OR coronary atheroscleros* OR COPD OR chronic obstructive lung disease* OR chronic obstructive pulmonary disease* OR chronic obstructive airway disease* OR asthma* OR diabet* OR hypertension OR high blood pressure OR stroke OR cancer OR neoplasm* OR arthritis OR chronic disease* OR chronic illness* OR chronically ill))
3. TS=(heart failure OR Coronary artery disease* OR ischemic heart disease* OR coronary arterioscleros* OR coronary atheroscleros* OR COPD OR chronic obstructive lung disease* OR chronic obstructive pulmonary disease* OR chronic obstructive airway disease* OR asthma* OR diabet* OR hypertension OR high blood pressure OR stroke OR cancer OR neoplasm* OR arthritis OR chronic disease* OR chronic illness* OR chronically ill)

1 AND 2 AND 3
