# Supplementary material for: Smart Devices for Older Adults Managing Chronic Disease: A Scoping Review
Source: JMIR Mhealth Uhealth. 2017 May 23;5(5):e69. doi: 10.2196/mhealth.7141 (PMC5461419; doi:10.2196/mhealth.7141)
Supplement: Multimedia Appendix 2 [file mhealth_v5i5e69_app2.pdf]

Description of the 51 included articles.

No.	Authors and Year (Reference)	Source Type	Study Design	Morbidity	Biometrics and PROMs	Types of self-management support	Types of decision support
1	Anglada-Martinez et al., 2016[28]	Journal article	Pre-experimental	Multi-morbidities: hypertension, dyslipidemia, heart failure, HIV	Medication adherence	Reminder, education, self-monitoring	Virtual
2	Arsand et al., 2010[29]	Journal article	Pre-experimental	T2DM	Blood glucose, step count	Self-monitoring	NA
3	Bengtsson, Kasperowski, Ring, & Kjellgren, 2014[30]	Journal article	Qualitative	Hypertension	NA	NA	NA
4	Bengtsson, Kjellgren, Hallberg, Lindwall, & Taft, 2016[31]	Journal article	Pre-experimental	Hypertension	Blood pressure, pulse, symptoms, well-being, medication adherence	Self-monitoring	NA
5	Clark et al., 2013[26]	Poster abstract	Mixed method	Heart failure	NA	NA	NA
6	Clark et al., 2015[27]	Journal article	Mixed method	Heart failure	NA	Education	NA
7	Cohen et al., 2015[32]	Poster abstract	Pre-experimental	T2DM	Blood glucose	NA	NA
8	Dang et al., 2014[33]	Poster abstract	Secondary data analysis	T2DM	Blood glucose, body weight	NA	NA
9	Ding, Karunanithi, Kanagasingam, Vignarajan, & Moodley, 2014[34]	Journal article	Pre-experimental	COPD	Temperature, pulse, oxygen saturation level	Self-monitoring, coaching	Telephone
10	Goldstein et al.,	Journal	RCT	Heart failure	Medication	Reminder, self-	NA

	2014[35]	article			adherence	monitoring	
11	Greenwood, Blozis, Young, Nesbitt, & Quinn, 2015[36]	Journal article	RCT	T2DM	Blood glucose	Self-monitoring, coaching, education, goal setting, reminder	Telephone, virtual
12	Hagglund et al., 2015[37]	Journal article	RCT	Heart failure	Body weight	Self-monitoring, automated feedback	DSS
13	Hallberg, Ranerup, & Kjellgren, 2016[38]	Journal article	Qualitative	Hypertension	NA	NA	NA
14	Hardinge et al., 2015[39]	Journal article	Observational	COPD	Pulse, oxygen saturation level	Self-monitoring, education, treatment plan	Telephone
15	Hunt, Sanderson, Ellison, C.W., & B.K., 2014[40]	Journal article	Quasi-experimental	T2DM	Blood glucose	Self-monitoring	NA
16	Johnston et al., 2013[41]	Journal article	Observational	COPD	Body weight, well-being	Self-monitoring	DSS, telephone
17	Karhula et al., 2015[42]	Journal article	RCT	Multi-morbidities: T2DM, heart failure	Blood glucose, blood pressure, step count, body weight	Self-monitoring, coaching	In-person
18	E. K. Kim et al., 2016[43]	Journal article	Pre-experimental	T2DM	Blood glucose, step count, diet	Self-monitoring, education, automated feedback, social support	DSS, telephone
19	E. K. Kim, Kwak, Park, Jang, & Cho, 2014[44]	Poster abstract	Pre-experimental	T2DM	blood glucose, step count	Self-monitoring	NA
20	H. S. Kim et al., 2014[45]	Journal article	Quasi-experimental	T2DM	Blood glucose, blood pressure	Self-monitoring	Virtual, telephone
21	Klasnja, Hartzler, Powell, & Pratt, 2011[46]	Conference proceeding	Quasi-experimental	Cancer	Symptoms	Self-monitoring, social support	NA
22	Liu et al., 2011[47]	Journal	RCT	Asthma	Peak expiratory	Self-monitoring,	DSS

		article			flow rate, symptoms	automated feedback, education	
23	Logan et al., 2012[48]	Journal article	RCT	Multi-morbidities: T2DM, hypertension	Blood pressure	Self-monitoring, automated feedback, reminder	DSS, virtual
24	Maguire et al., 2015[49]	Journal article	Mixed method	Cancer	Symptoms	Self-monitoring, automated feedback	DSS, telephone
25	Mira et al., 2014[50]	Journal article	RCT	Multi-morbidities	Medication adherence	Self-monitoring	NA
26	Nes et al., 2012[51]	Journal article	Qualitative	T2DM	Blood glucose, symptoms	Self-monitoring, education, automated feedback, reminder	NA
27	Patel et al., 2013[52]	Journal article	Quasi-experimental	Hypertension	Medication adherence	Self-monitoring, reminder, education	NA
28	Pellegrini et al., 2015[53]	Journal article	Pre-experimental	T2DM	Step count	Self-monitoring, automated feedback	NA
29	Quinn, Khokhar, Weed, Barr, & Gruber-Baldini, 2015[54]	Journal article	Pre-experimental	T2DM	Blood glucose, diet, medication adherence, exercise	Self-monitoring, automated feedback, coaching, education	DSS
30	Quinn et al., 2016[55]	Journal article	Secondary data analysis	T2DM	NA	NA	NA
31	Quinn et al., 2011[56]	Journal article	RCT	T2DM	Blood glucose, diet, medication adherence	Self-monitoring, coaching, automated feedback, education	DSS, virtual
32	Schreier et al., 2012[57]	Journal article	Pre-experimental	Multi-morbidities: Type 1 and 2 diabetes	Blood glucose, blood pressure, body weight, well-being	Self-monitoring	NA
33	Seto et al., 2010[58]	Journal article	Qualitative	Heart failure	NA	NA	NA
34	Seto et al., 2012[59]	Journal article	RCT	Heart failure	Blood pressure, pulse, body weight,	Self-monitoring, automated feedback,	DSS, telephone

					ECG	reminder	
35	Shinohara et al., 2013[60]	Conference proceeding	Pre-experimental	Rheumatoid arthritis	Gait, medication adherence, well-being	Self-monitoring	NA
36	Stahlberg et al., 2014[61]	Poster abstract	Pre-experimental	Heart failure	Blood glucose, body weight	Self-monitoring	NA
37	Tabak, Akker, & Hermens, 2014[62]	Journal article	Secondary data analysis	COPD	NA	NA	NA
38	Tabak, Vollenbroek-Hutten, van der Valk, van der Palen, & Hermens, 2014[63]	Journal article	RCT	COPD	Step count	Self-monitoring, goal setting, automated feedback	DSS
39	Tatara et al., 2013[64]	Journal article	Mixed method	T2DM	NA	NA	NA
40	Triantafyllidis et al., 2015[65]	Journal article	Pre-experimental	Heart failure	Blood pressure, pulse, oxygen level, body weight, symptoms	Self-monitoring, education	Virtual
41	van der Weegen et al., 2015[66]	Journal article	RCT	Multi-morbidities: COPD or T2DM	Step count, exercise	Self-monitoring, coaching, goal setting, education, automated feedback	Virtual
42	Verwey et al., 2014a[67]	Journal article	Protocol	Multi-morbidities: COPD or T2DM	NA	NA	NA
43	Verwey et al., 2014b[68]	Journal article	Mixed method	Multi-morbidities: COPD or T2DM	Step count	Self-monitoring, goal setting, automated feedback, coaching	In-person
44	Vuorinen et al., 2014[69]	Journal article	RCT	Heart failure	Blood pressure, pulse, body weight, symptoms	Self-monitoring, automated feedback	DSS, telephone
45	Waki et al., 2015[70]	Journal article	Pre-experimental	T2DM	Blood glucose, blood pressure,	Self-monitoring, automated feedback	DSS

					step count, body weight, diet, exercise, photo		
46	Waki et al., 2014[71]	Journal article	RCT	T2DM	Blood glucose, blood pressure, step count, body weight, diet, exercise, photo	Self-monitoring, automated feedback	NA
47	Wayne & Ritvo, 2014[72]	Journal article	Pre-experimental	T2DM	Blood glucose, blood pressure, body weight, diet, exercise, symptoms	Self-monitoring, coaching, goal setting	Virtual
48	Williams, Price, Hardinge, Tarassenko, & Farmer, 2014[73]	Journal article	Qualitative	COPD	NA	NA	NA
49	Wolf, Olsson, Swedberg, & Ekman, 2012[74]	Poster abstract	Qualitative	Heart failure	Body weight, symptoms	Self-monitoring	NA
50	Wood, Alley, Baer, & Johnson, 2015[75]	Journal article	Pre-experimental	T2DM	NA	Education	NA
51	Wu et al., 2015 [76]	Journal article	Quasi-experimental	Cancer	Photo, symptoms	Self-monitoring	Telephone
